# Cell-free fat extract regulates oxidative stress and alleviates Th2-mediated inflammation in atopic dermatitis

**DOI:** 10.3389/fbioe.2024.1373419

**Published:** 2024-04-26

**Authors:** Zexin Fu, Qinhao Gu, Lu Wang, Lulu Chen, Liuyi Zhou, Qiang Jin, Ting Li, Ye Zhao, Sufan Wu, Xuejiao Luo, Tingting Jin, Chengrui Guo

**Affiliations:** ^1^ The Second Clinical Medical College, Zhejiang Chinese Medical University, Hangzhou, China; ^2^ Center for Plastic and Reconstructive Surgery, Department of Plastic and Reconstructive Surgery, Zhejiang Provincial People’s Hospital, Affiliated People’s Hospital, Hangzhou Medical College, Hangzhou, China; ^3^ Hangzhou Normal University Division of Health Sciences, Hangzhou, China; ^4^ Department of Dermatology, The Affiliated Hospital of The NCO School, The Army Medical University, Shijiazhuang, China

**Keywords:** cell-free fat extract, atopic dermatitis, oxidative stress, Th2 inflammatory, DNCB

## Abstract

Atopic dermatitis (AD) is a common inflammatory skin disease that significantly affects patients’ quality of life. This study aimed to evaluate the therapeutic potential of cell-free fat extract (FE) in AD. In this study, the therapeutic effect of DNCB-induced AD mouse models was investigated. Dermatitis scores and transepidermal water loss (TEWL) were recorded to evaluate the severity of dermatitis. Histological analysis and cytokines measurement were conducted to assess the therapeutic effect. Additionally, the ability of FE to protect cells from ROS-induced damage and its ROS scavenging capacity both *in vitro* and *in vivo* were investigated. Furthermore, we performed Th1/2 cell differentiation with and without FE to elucidate the underlying therapeutic mechanism. FE reduced apoptosis and cell death of HaCat cells exposed to oxidative stress. Moreover, FE exhibited concentration-dependent antioxidant activity and scavenged ROS both *in vitro* and *vivo*. Treatment with FE alleviated AD symptoms in mice, as evidenced by improved TEWL, restored epidermis thickness, reduced mast cell infiltration, decreased DNA oxidative damage and lower inflammatory cytokines like IFN-γ, IL-4, and IL-13. FE also inhibited the differentiation of Th2 cells *in vitro*. Our findings indicate that FE regulates oxidative stress and mitigates Th2-mediated inflammation in atopic dermatitis by inhibiting Th2 cell differentiation, suggesting that FE has the potential as a future treatment option for AD.

## 1 Introduction

Atopic dermatitis (AD) is a chronically inflammatory eczematous skin disorder characterized by various clinical manifestations, including thickened, dried, cracked skin and pruritus. Although the pathogenesis of AD remains unclear, multiple studies have suggested that excessive oxidative stress and immunopathology are closely related to AD (Narla and Silverberg, 2020; Sroka-Tomaszewska and Trzeciak, 2021; Galiniak et al., 2022). Th2-mediated inflammation is a characteristic immune response in AD. Due to mutation in the filaggrin gene (FLG), plenty of immunogens enter the damaged skin barrier and attract Th2 cells to produce a stream of inflammatory cytokines such as interleukin (IL)-4 and IL-13 (Eyerich and Novak, 2013). Ultimately, these cytokines induce excessive oxidative stress and further disrupt skin barrier. Oxidative stress is due to an imbalance in production and elimination of reactive oxygen species (ROS). Excessive ROS production leads to oxidative damage to nucleic acid, proteins and lipid membranes (Santos et al., 2018). For instance, DNA oxidative damage marker 8-hydroxy-2′-deoxyguanosine (8-OHdG) was found to be elevated in AD patients compared to healthy individuals (Chen et al., 2020). Additionally, AD patients have been reported to exhibit higher levels of advanced protein oxidative products and lower levels of antioxidants (Galiniak et al., 2022). The relationship between Th2 cell-mediated inflammation and ROS forms a cycle of mutual promotion in which excessive ROS further enhances IL-4 secretion, leading to the differentiation of naïve T cells into Th2 cells. Therefore, targeting ROS-scavenging and Th2-mediated cytokines would be the most promising therapeutic targets in AD research (Ji and Li, 2016; Schneider, 2017; Onodera et al., 2023).

According to guidelines, topical corticosteroids (TCS) remain the first-line treatment for acute period control and chronic phase long-term remission (Chin J Dermatol, 2020; Katoh et al., 2020; Ständer, 2021; Lax et al., 2022). TCS can effectively inhibit overwhelming inflammation. However, prolonged and extensive use of corticosteroids can result in adverse local and systemic reactions (Chiricozzi et al., 2020). Besides, there is an emergence of Th2-targeted biologics approved for AD patients, such as monoclonal IL4 receptor antibodies, phosphodiesterase-4 inhibitors, and JAK inhibitors (Ständer, 2021). Dupilumab, the monoclonal IL-4/-13 antibody, specifically binds to IL-4R and inhibits the secretion of IL-4 and IL-13 to block Th2 cell-mediated inflammation (Beck et al., 2014; Paller et al., 2020). These agents lack specificity in targeting immune dysregulation in AD, potentially leading to severe adverse events, including allergic conjunctivitis, thromboembolism, and liver dysfunction (Ständer, 2021). Apart from its side effects, it presents difficulties in being utilized as a daily management medication for AD due to the cost and approval of only few countries. Hence, there is a critical necessity to create new, accessible alternative treatments to control and manage AD.

Human adipose tissue is regarded as a “stem cell bank” because it contains a significant amount of adipose tissue-derived stem cells (ADSCs) with anti-inflammatory, multiple differentiation, and immune regulation potential. ADSCs have been reported to have immunomodulatory effects in the treatment of AD (Guan et al., 2022). The therapeutic effect of ADSCs is not due to the potential ability of differentiation or regenerative, but rather to their secretion of biologically active soluble substances (Qazi et al., 2017). Delivery of therapeutic factors instead of stem cell transplantation has recently been considered a superior alternative strategy. The stromal vascular fraction (SVF), enriched with ADSCs, is isolated from centrifugal emulsification fat and has found widespread application in tissue regeneration. Based on the preparation of SVF, we further integrate the power of mechanical and freeze-thaw cycles to fully disrupt cells and release biologically active soluble substances to obtain the liquid cell-free FE. Zhang and his colleague found that the FE produced by the mechanical and freeze-thaw methods and the factors secreted by ADSCs share a large number of collections via proteomic studies (Yu et al., 2018). The cell-free nature of FE allows it to avoid some potential risks associated with cell transplantation, such as immune response and tumor formation. Furthermore, FE can be prepared and stored more easily without the need for complex cell culture techniques. Theoretically, the use of cell-free FE should not elicit immune rejection reactions, making FE potentially lower risk in clinical applications compared to ADSCs. However, its therapeutic effect on AD has not been reported and remains unclear.

**FIGURE 1:**
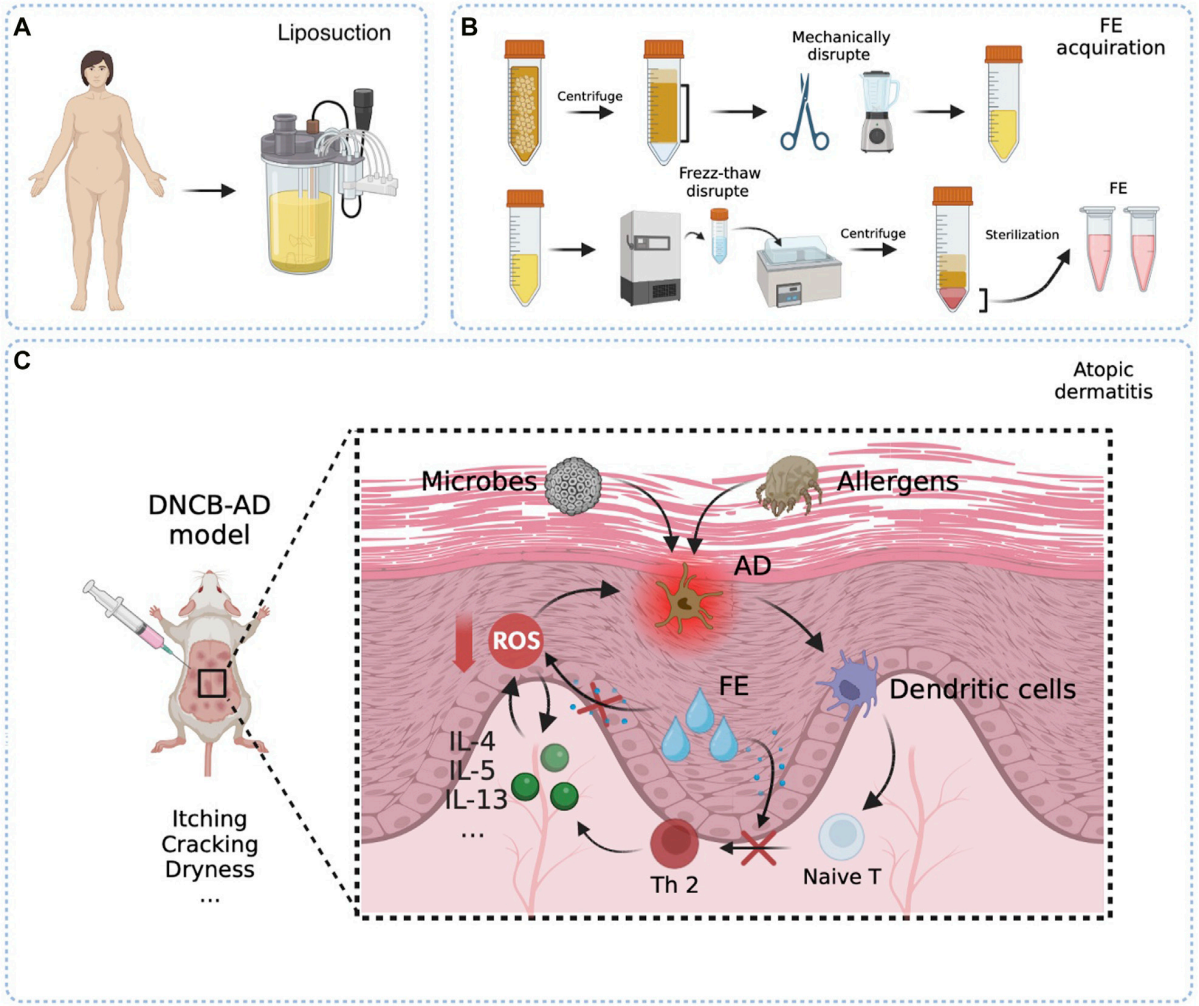
Schematic presentation of the easy fabrication process of FE and therapeutic mechanism in the DNCB-AD model. **(A)** Raw material fat obtained from liposuction surgery. **(B)** Raw fats were centrifuged to remove oil and water. The FE liquid is obtained by mechanical and freeze-thaw methods to fully destroy the tissues and cells. **(C)** Repeated and multiple applications of the hapten DNCB disrupted skin barrier and induced a Th2-biased immune response that well mimics human AD. The allergic agents penetrate through the damaged skin barrier, become recognized by antigen-presenting cells, and stimulate an differentiation into Th2 cells. In turn, Th2 cells secrete pertinent inflammatory factors and widespread ROS, ultimately triggering dermatitis symptoms and contributing to the further degradation of the skin barrier. FE would truncate the circulatory relationship between Th2 cell-mediated inflammation and the promotion of ROS.

This study aimed to broaden the understanding of the therapeutic effect and mechanism of FE in AD. Our study revealed for the first time that FE can clear ROS and Th1/2 cell balance immunoregulation and improve AD clinical symptoms (Figure 1). Herein, FE may help manage and treat AD due to its ROS-scavenging and immunomodulatory properties.

## 2 Materials and methods

### 2.1 FE preparation

FE was extracted from fat tissue following the method described previously by Xu G-Y. et al. (2022). Briefly, after being washed thrice with saline, lipoaspirate was centrifuged at 1,200 *g* to remove oil and water. A high-speed blender was then used to mechanically and thoroughly emulsify washed adipose tissue. The emulsified fat liquid went through a process of freezing at −80°C followed by rapid thawing for disruption. The fat liquid was centrifuged at 1200 g to obtain the aqueous layer at the bottom. Finally, the reserved clear yellow liquid was filtered three times through a 0.22 μm filter and stored at −80°C. The protein concentrations of FE were measured using the Enhanced BCA Protein Assay Kit (Beyotime, Shanghai, China). All patients had signed informed consent.

### 2.2 Cell culture

HaCat cells (Human Keratinocytes Cells; Nanjing Saihongrui Biotechnology Co., Ltd.) and HUVEC (Human Umbilical Vein Endothelial Cells, Generously donated by Dr. Yuewei Chen of Zhejiang University in Hangzhou, China) were used for investigation. All these cell lines were cultured in Dulbecco’s modified Eagle’s medium (DMEM) (Dalian Meilun Biotechnology Co., Ltd., Meilunbio) supplemented with 10% fetal bovine serum (Dalian Meilun Biotechnology Co., Ltd., Meilunbio) and 1% P/S. The cells were maintained at 37°C in a humidified atmosphere containing 5% CO_2_.

### 2.3 Cell viability assay in a high ROS environment

HaCat cells were seeded at a density of 3 × 10^4^ ml^−1^ in 96-well plates. After 24 h, HaCat cells were treated with 800 μM hydrogen peroxide (H_2_O_2_) to mimic extracellular high ROS environment and co-treated with low (50 μg/mL, L-FE), medium (200 μg/mL, M-FE), and high (500 μg/mL, H-FE) concentrations of FE. Subsequently, cell proliferation rates were determined using the cell counting kit-8 (CCK-8, Dalian Meilun Biotechnology Co., Ltd., Meilunbio), and living and dead cells were identified using Calcein/Propidium Iodide (PI) Cell Viability/Cytotoxicity Assay Kit (Beyotime, Shanghai, China). A fluorescence microscope (EVOS M700, Thermo Fisher, United States) was used to calculate cell viability with the following formula:

$$\text{Cell viability }\left(\%\right)=\frac{\text{Number}\;\text{of}\;\text{Calcein}+\text{Cells}}{\text{Number}\;\text{of}\;\text{Calcein}+\text{Cells}+\text{Number}\;\text{of}\;\text{PI}+\text{Cells}}\times 100$$

### 2.4 Cell apoptosis in high ROS environment

The cell apoptosis of HaCat cells was determined using a Cell Cycle and Apoptosis Analysis Kit (Beyotime, Shanghai, China). After the same treatments, cells were collected, resuspended, and incubated with 5 μL of Annexin V and 5 μL of PI. After 30 min incubation in the dark, apoptosis was performed by quantification of the sub-G1 peak via a Novocyte flow cytometer. The final test data were analyzed using NovoExpress (Agilent Technologies, Inc., United States).

### 2.5 Measurement of intracellular ROS

Intracellular ROS generation was assessed using ROS Assay Kit (Beyotime, Shanghai, China). After incubation with 60 μM DNCB (237329, Merck, Shanghai, China) for 5–6 h, and co-treatment with low (50 μg/mL, L-FE), medium (200 μg/mL, M-FE), and high (500 μg/mL, H-FE) concentrations of FE, all group cells were stained with 10 μM 1 × 2′, 7′-dichlorofluorescein-diacetate (DCFH-DA) at 37 °C for 30 min and then scanned under a fluorescence microscope EVOS M700, Thermo Fisher, United States. Cellular DCF^+^ fluorescence was also quantified using a Novocyte flow cytometer and analyzed using NovoExpress (Agilent Technologies, Inc., United States).

### 2.6 Superoxide dismutase (SOD) mimicking properties of FE

The SOD-mimicking properties of FE were measured using a Total Superoxide Dismutase Assay Kit with WST-8 (Beyotime, China). Zero, low, medium, and high concentrations of FE were added to 96-well plates along with 160 μlof WST-8 working solution (including 1 μL of xanthine oxidase (XO) solution and 8 ul WST-8). The system was then incubated for 30 min at 37 °C. The absorbance of WST-8 formazan was measured at 450 nm using a microplate reader.

### 2.7 T cell isolation and Th2 differentiation *in vitro*

Spleens were isolated from selectedmouse strains and placed in ice-cold 2% FBS for naïve T cell collection. The organs were prepared by pulverizing them through a 100 μm filter. Lymphocytes were filtered through a 40 μm filter and resuspended with MojoSort™ Buffer (Biolegend San Diego, CA, United States). Naïve CD4^+^ T cells were isolated using MojoSort™ Mouse CD4 Naïve T Cell Isolation Kit (Biolegend San Diego, CA, United States), according to the manufacturer’s instructions. Naïve CD4^+^ T cells were resuspended with Roswell Park Memorial Institute (RPMI) 1,640 (Dalian Meilun Biotechnology Co., Ltd., meilunbio) to a final concentration of 1 × 10^6^/mL. Cells at a density of 10^6^/mL were cultured on 24well-plates pretreated with 5 μg/mL of anti-mouse CD3 (clone 145-2C11) and 10 μg/mL of anti-mouse CD28 (clone 37.51).

After the abovementioned stimulation for 24 h, the CD4^+^ T Cells were washed with phosphate-buffered saline (PBS 1×) and cultured for 3 days with 10 ng/mL of rIL-2, 10 ng/mL of rIL-4, and 10 μg/mL of anti-mouse IFN-γ for Th2-polarization. Simultaneously, zero, low, medium, and high concentrations of FE were added to 24-well plates to inhibit differentiation. All recombinant cytokines and antibodies against cytokine were purchased from BioLegend San Diego, CA, United States.

### 2.8 Flow cytometric analysis of intracellular cytokine synthesis

All cells cultured with Th2 polarizing condition were harvested, washed twice with PBS, and activated with Cell Activation Cocktail (Phorbol-12-Myristate 13-Acetate (PMA, 40.5 µM), ionomycin (669.3 µM) for 6h, and Brefeldin A (2.5 mg/mL), BioLegend San Diego, CA, United States). Then, cells were harvested, washed twice with PBS, and incubated for 30 min at room temperature in the dark with anti-CD3-BV421 and anti-CD4-FITC. After being washed with PBS, cells were fixed and permeabilized for 20 min at room temperature with Cyto-Fast™ Fix/Perm Buffer (Biolegend San Diego, CA, United States). After another PBS wash, cells were incubated at room temperature for 30 min with anti-IFN-γ-PE/Cyanine7 and anti-IL-4-PE (Biolegend San Diego, CA, United States). Finally, the cells were washed with PBS, and 2 × 10^5^ cells were acquired by a Novocyte flow cytometer and analyzed with NovoExpress (Agilent Technologies, Inc., United States).

### 2.9 Animals

In total, 65 female Balc/b mice aged 6—8 weeks were used for the *in vivo* study (Shanghai SLAC Laboratory Animal Co. Ltd., Shanghai, China). All animal experimental procedures were approved by the Laboratory animal management and ethics committee of Zhejiang Provincial People’s Hospital.

### 2.10 *In vivo* therapeutic effect of FE with H_2_O_2_ sensitization

We used inflamed skin wounds induced by attaching gauze with 8% H_2_O_2_ for 2 days. After removing the H_2_O_2_ gauze, the damaged skin area was injected intradermally with 62.5 μL FE or saline solution once per week (low FE group) or twice per week (high FE group). According to the plan, the skin lesions of mice were photographed. Finally, all mice were sacrificed by CO_2_ inhalation.

### 2.11 *In vivo* therapeutic effect of FE on DNCB-induced AD mouse model

DNCB-induced AD mouse model was created as previously described (Gilhar et al., 2021; Saini et al., 2022). DNCB was dissolved in a 3:1 mixture of acetone and olive oil to induce AD on the back skin. Except for the normal group, 150 μL of 1% DNCB was applied to the dorsal skin of each mouse during the initial weeks. After a latency period of 1 week, 100 μL of 0.5% DNCB was administered for 2 weeks as the second sensitization. During the treatment period, 62.5 μL FE was injected intracutaneously with a 30G needle once a week (low FE group) or twice a week (high FE group) into skin lesions. The negative group was injected with an aseptic saline solution, while the positive group was treated with 0.05% Desonide Cream (glucocorticoid, GC) (Chongqing Banghua Pharmaceutical Co. Ltd. China) and sham injection twice a week. Additionally, 100 μL of 0.5% DNCB was applied the day after treatment to mimic continuous allergen exposure. According to the plan, the skin lesions of mice were photographed, observed, and scored, and transepidermal water loss (TEWL) was measured. △TEWL was calculated using the following formula:

$$△\text{TEWL}=\left[{\text{TEWL}}_{\left(\text{before}\;\text{treat},W4\right)}‐{\text{TEWL}}_{\left(\text{baseline}\right)}\right]‐\left[{\text{TEWL}}_{\left(\text{after}\;\text{treatment},W6\right)}‐\;{\text{TEWL}}_{\left(\text{baseline}\right)}\right]$$

The dermatitis score was adjusted according to the SCORAD (scoring atopic dermatitis index) and measured as the sum of scores graded as 0 (no typical symptoms), 1 (mild), 2 (moderate), or 3 (severe) for each of the four characteristic symptoms of atopic dermatitis (erythema/hemorrhage, scarring/exudation, edema, excoriation/erosion).

### 2.12 Histological analysis

The sensitized skin was fixed with paraformaldehyde, embedded in paraffin wax, stained with hematoxylin and eosin, and toluidine blue O for histological analysis. The epidermal thickness and number of mast cells were evaluated in five fields per section. For immunofluorescent staining, frozen skin sections were incubated with 8‐OHdG (sc-393871, Santa Cruz, CA, United States) at 37 °C in the dark for 1 h. The sections were then incubated with DAPI (36308ES20, Yeasen, China) staining for 30 min at room temperature in the dark. Immunofluorescence was detected using a fluorescence microscope (EVOS M700, Thermo Fisher, United States). The number of positive cells per field was determined in five fields per section and five sections per femoral head. Quantitative analysis was done using ImageJ software.

### 2.13 Th1/2 cells balance in FE treating AD-mouse model

Mice treated with FE or topical corticosteroids were sacrificed, and their skin lesions were collected. The skin tissue was cut into small pieces and homogenized using a 1 mm φ grinding bead mill homogenization. After 10 min of Centrifugation at 500 *g* for 10 min, the supernatant was collected for subsequent detection. The levels of IL-4, IL-13, IL-17A, IFN-γ, and TNF-α were measured by multiplex secretome analysis (ABplex Mouse 5-Plex Custom panel), according to manufacturer’s instructions and with ABclonal Technology Co., Ltd.

### 2.14 Statistical analyses

Quantitative data are presented as the mean ± standard deviation. Differences between the groups were analyzed using one-way analysis of variance (ANOVA) or non-parametric tests. Statistical analyses were performed using IBM SPSS software (SPSS Statistics V22, IBM Corporation, United States). Values of *p* < 0.05 were considered statistically significant. *p < 0.05, **p < 0.01, ***p < 0.001, ****p < 0.0001.

## 3 Results

### 3.1 FE protects HaCat cells from ROS-induced damage

**FIGURE 2:**
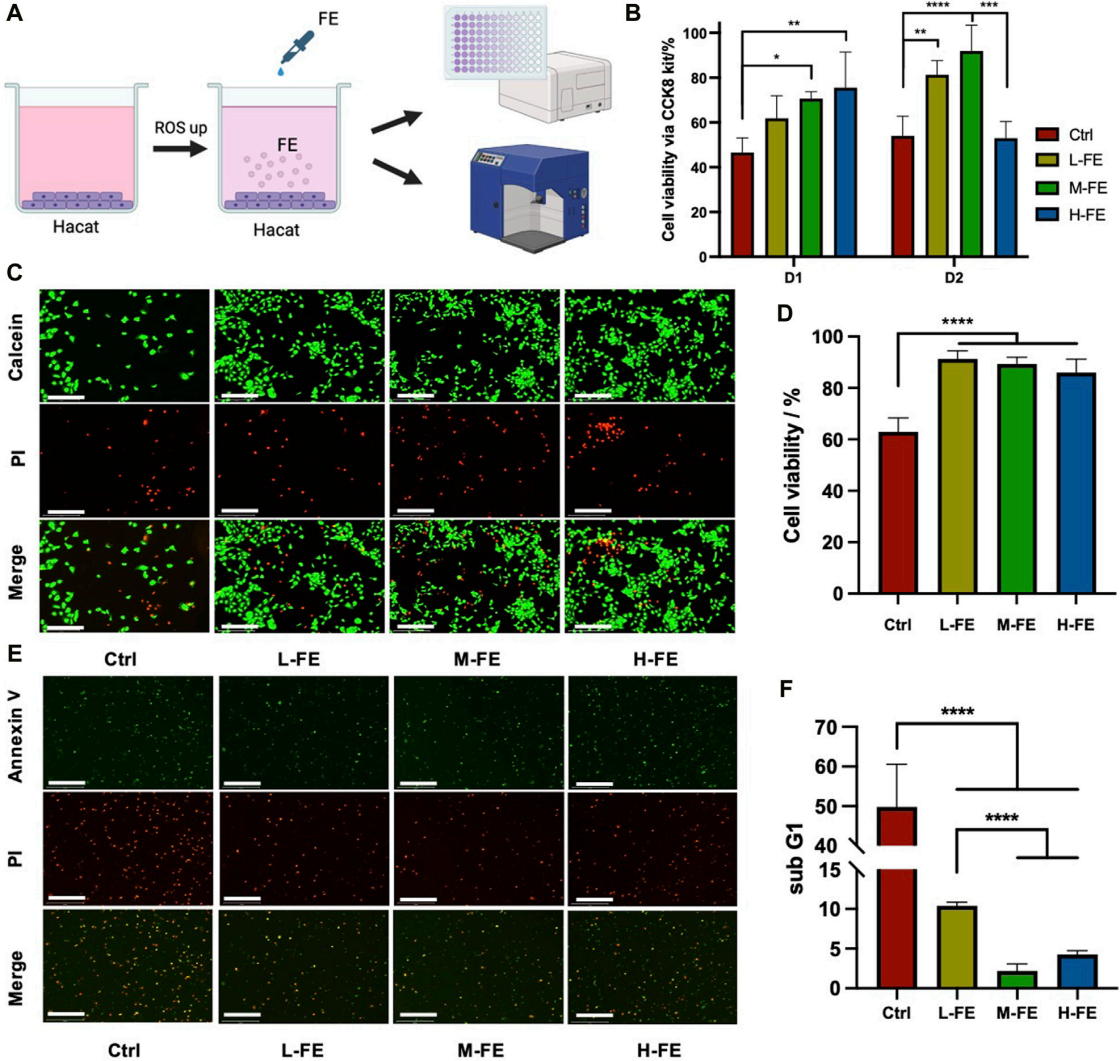
FE increases cell viability in a high ROS extracellular environment. **(A)** Schematic images of *in vitro* experiments with high ROS environment. **(B)** CCK8 kit was used to detect cell viability after incubation with an extracellular high ROS environment in different FE concentrations. **(C)** Live/dead stains showed a cytoprotective effect of FE under a highly oxidative medium by H2O2 (green: Calcein, live cell; red: Propidium iodide, dead cell). Scale bar = 275 μm. **(D)** Quantitative analysis of live/dead assay fluorescence. **(E)** Early apoptosis level in a high ROS environment was observed by fluorescence microscope. The HaCat cells were in suspended status after digestion. (green: Annexin V, red: propodium iodide). Scale bar = 275 μm. **(F)** The proportion of apoptotic cells was quantitatively analyzed through the subG1 peak. (**p < 0.01, ***p < 0.001, ****p < 0.0001).

To assess the protective effects of FE against ROS, cultured HaCat cells were exposed to a high concentration of H_2_O_2_ and then rescued with different concentrations of FE (Figure 2A). H_2_O_2_ was added in the medium to induce a high ROS extracellular environment, injuring over half of HaCat cells. Data using CCK8 kit showed a dose-dependent recovery of cell viability with increasing FE concentration (Figure 2B). A similar result was observed in calcein/PI staining, revealing that FE effectively rescued cell viability. With the addition of FE, the number of living cells increased significantly in every high-magnification field. Cell viability also increased considerably compared to the ROS control group (Figures 2C, D). As extracellular ROS can induce apoptosis (Zhang et al., 2015), the degree of apoptosis reflects ROS-induced cytotoxicity. The fluorescence results shown by the apoptosis kit observed a decrease in the number of green + cells after FE intervention (Figure 2E), and for more accurate quantification, flow cytometry analyzed the Sub-G1 peaks, which reflected the proportion of apoptotic cells (Haridevamuthu et al., 2023). The results showed that L-, H- and M-FE all reduced apoptosis caused by high ROS (Figure 2F). But interestingly the apoptosis rate was increased in the H-FE group compared to M-FE (this finding seems to be similar to the CCK8 results of D2 in Figure 2B).

### 3.2 ROS scavenging ability of FE

**FIGURE 3:**
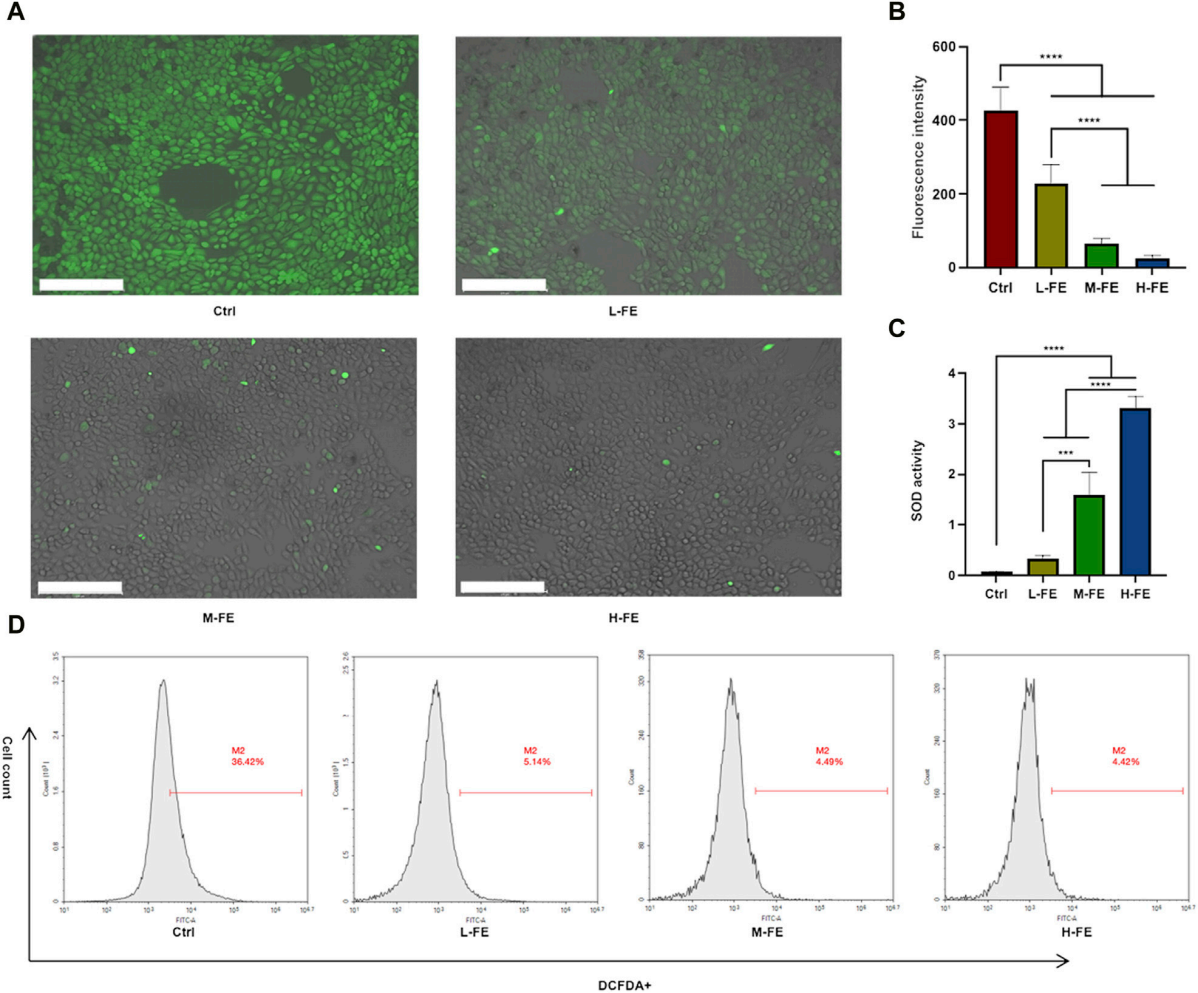
FE increases cell viability via ROS scavenging in a high ROS extracellular environment. **(A)** Intracellular ROS was measured via DCF-DA staining, indicating that FE decreased the level of ROS in a dose-dependent way. Image is shown by a merge of fluorescence and bright field. Scale bar = 275 μm. **(B)** Quantitative analysis of DCFDA + positive fluorescence intensity. **(C)** SOD-mimicking activity in FE with various concentrations. **(D)**. Flow cytometry analysis of intracellular ROS levels of HaCat cells after incubating with FE. (**p < 0.01, ***p < 0.001, ****p < 0.0001).

To determine how FE increases cell viability under a high ROS environment, whether by promoting cell proliferation or affecting the level of ROS. The potential ROS scavenging ability of FE was analyzed. Without FE, HaCat cells’ green fluorescent signal increased markedly, and the intracellular ROS fluorescence intensity decreased significantly (Figures 3A, B). We further performed a quantitative analysis of intracellular ROS levels using flow cytometry. The results demonstrated that FE eliminated intracellular ROS levels from high ROS-treated HaCat cells, favoring positive ROS scavenging of FE (Figure 3D).

SOD is an important antioxidant enzyme in cells that reduces ROS accumulation. The ROS-scavenging properties of FE were also tested by SOD activity. It was found that FE efficiently removed ROS in a concentration-dependent manner (Figure 3C). The above results suggested that FE mimicking SOD increased cell viability by reducing ROS in a high ROS environment.

### 3.3 Therapeutic efficacy of FE in H_2_O_2_ damage mice skin

**FIGURE 4:**
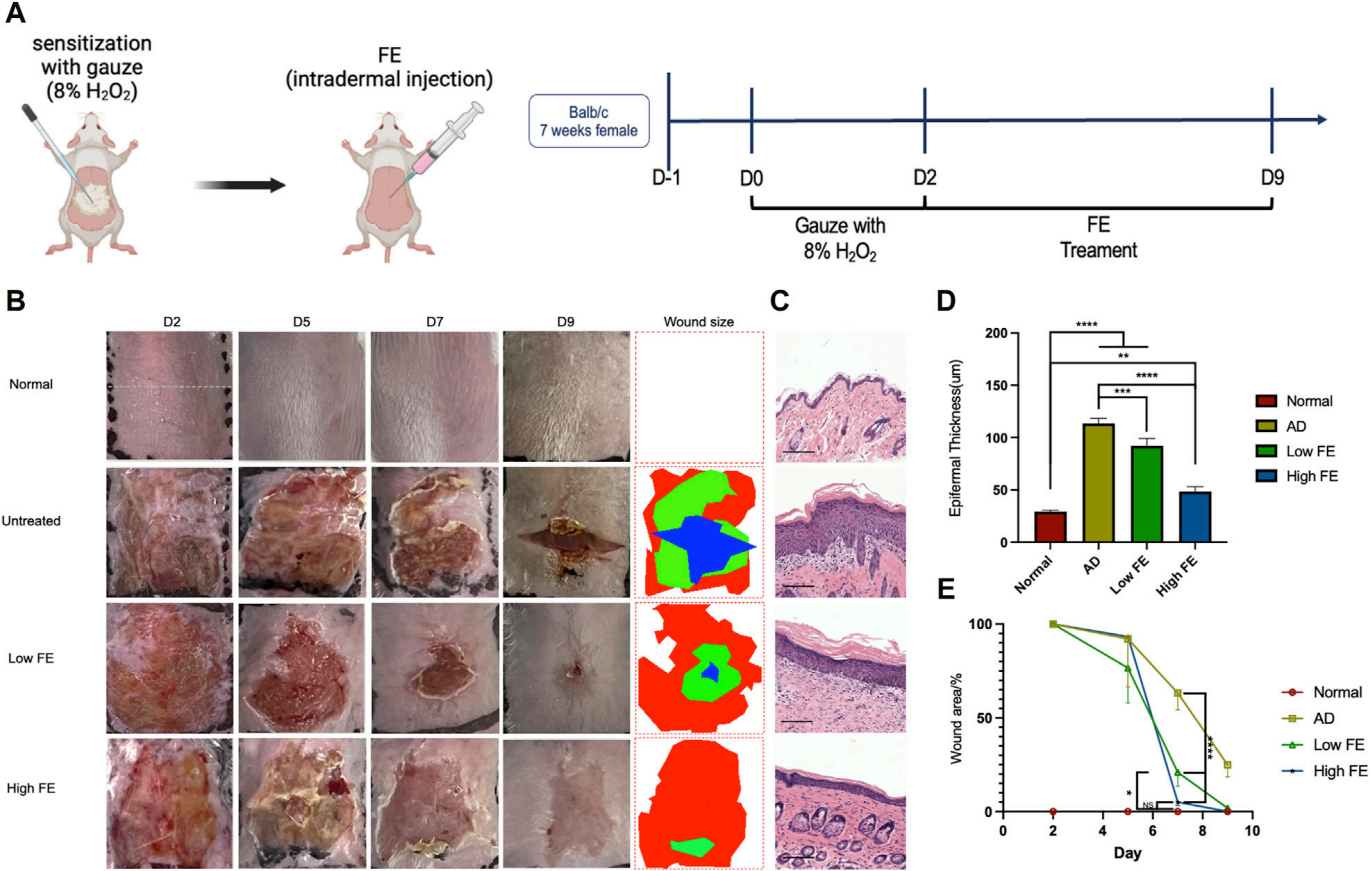
FE treated H_2_O_2_-induced inflammatory skin model *in vivo*. **(A)** Schema and timeline for *in vivo* intradermal ROS experiments with H_2_O_2_ and the subsequent treatments by FE. **(B)** Representative photographs of skin lesions in each group after treatments. The rightmost column is wound size measurements based on the photographs. **(C)** Hematoxylin and eosin stain of mouse skin tissue sections after treatment, showing the thickness of the epidermis became thinner after FE treatment (scale bar = 125 μm). **(D)** Measurements of epidermal thickness for each group. **(E)** Quantitative analysis of wound size. (**p < 0.01, ***p < 0.001, ****p < 0.0001).

To assess the anti-inflammatory effects of FE *in vivo*, we used a mouse model treated with H_2_O_2_ to mimic inflammatory skin conditions characterized by elevated ROS levels and resembling features observed in atopic dermatitis (AD) (Figure 4A) (Trenam et al., 1992; Izu et al., 2000). It was observed that FE treatment reduced skin lesion area, and the high FE group healed better than the low FE group (Figure 4B). The wound size observed in the high FE-treated group on day seven was much smaller than the untreated and low FE-treated group (Figures 4B, E) and recovered faster than the other groups.

After sacrificing the mice on day nine, the H&E stain showed that the high FE-treated group had significantly thinner epidermal thickness than other groups (Figures 4C, D). In conclusion, FE with ROS scavenging ability can alleviate the adverse outcomes of dermatitis induced by H_2_O_2_ in a mouse model.

### 3.4 Therapeutic efficacy of FE in DNCB-induced AD mice model

The above data showed that FE has a potential ROS scavenging ability *in vivo* and *in vitro* and can alleviate oxidative stress-induced skin damage. We further used the DNCB-induced AD model to detect the therapeutic effect of FE.

**FIGURE 5:**
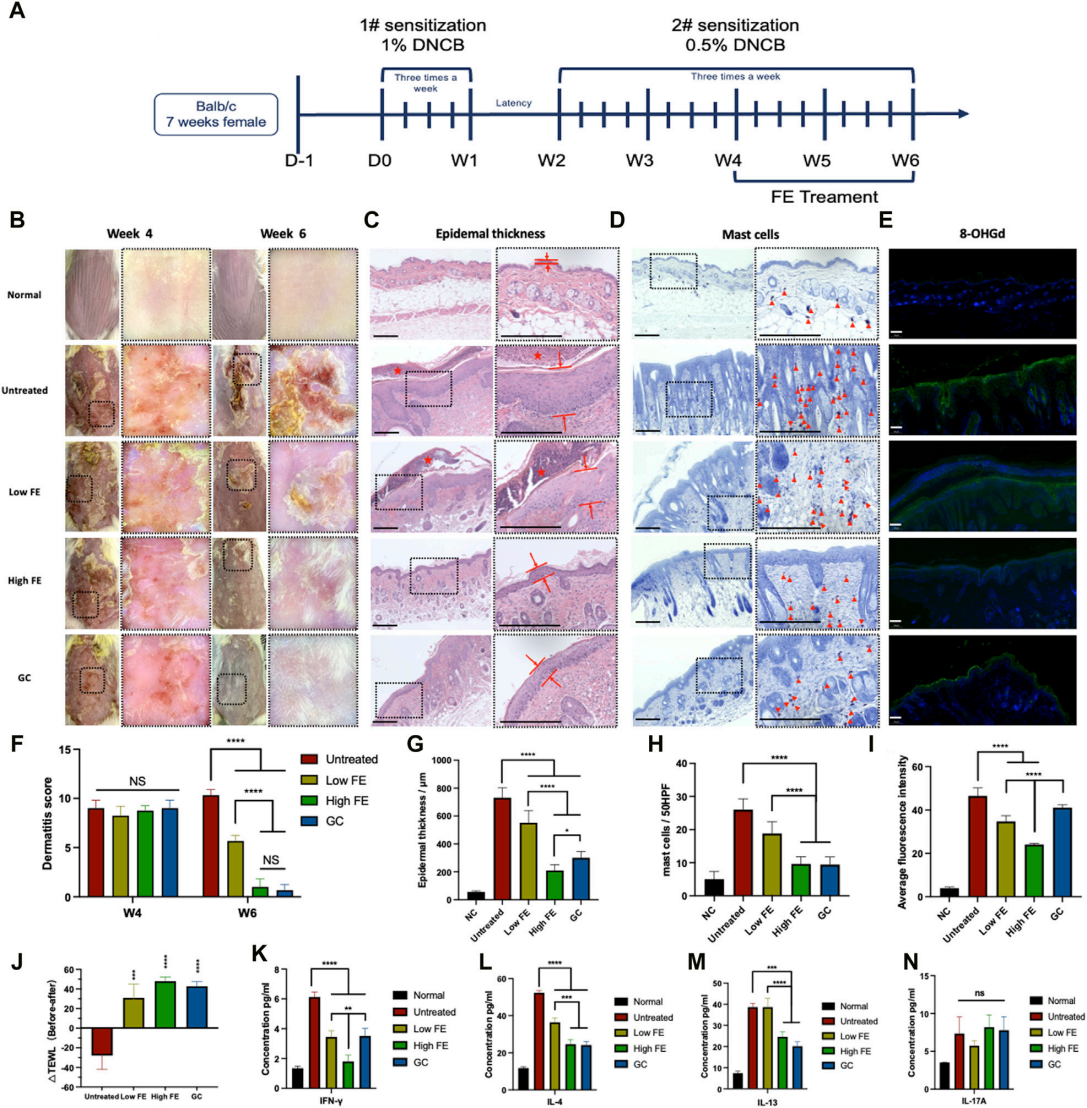
FE treated DNCB-induced AD model *in vivo*. **(A)** Timeline for *in vivo* experiments of AD induction by sensitization with DNCB and the following treatments by intracutaneous injection of FE. **(B)** Representatively general and dermatoscopic photographs of the dorsal skin of each group. **(C)** H&E stained skin tissue’s enlarged image (right) to evaluate epidermal thickness. The distance between red lines represents the epidermal thickness. The red star indicates scabs. Scale bar = 1,250 μm. **(D)** Histology of skin sections stained with toluidine blue and an enlarged image to analyze the number of mast cells (right). The red triangle icons indicate the mast cells. Scale bar = 1,250 μm. **(E)** Representative fluorescent immunostaining images for ROS damage marker 8-OHdG with DAPI staining from the skin lesion tissue of each group retrieved at W6 (green: 8-OHdG, blue: DAPI). Scale bar = 100 μm **(F)** Dermatitis score measurements and **(J)** TEWL measurements of each group conducted 6 weeks before and after treatment. The quantitative analysis of **(G)** epidermal thickness, **(H)** number of mast cells, **(I)** the intensity of stained 8-OhdG. The concentrations of **(K)** IFN-γ, **(L)** IL-4, **(M)** IL-13, and **(N)** IL-17A in skin lesion tissue retrieved from each group at the end of the 6th week. (*p < 0.05, **p < 0.01, ***p < 0.001, ****p < 0.0001).

DNCB, an incomplete antigen used to prepare AD animal models, interacts with skin protein to form complexes (Kim et al., 2019). The complexes would then be recognized by antigen-presenting cells (e.g., Langerhans cells and dermal dendritic cells) to activate Th2 and mast cells. We performed the modeling procedure described above (Figure 5A), which induced Th2-mediated immune dysfunction mimicking AD. At week four (W4), all groups had skin wounds with scarring and erosion, and their dermatoscopy showed obvious and typical dermatitis symptoms, indicating that AD was well-induced in the skin at W4 (Figure 5B, W4). After 2 weeks of treatment, the general and dermatoscopic photographs of the untreated group (positive control) showed severe skin barrier damage with xerosis, erythema, scarring, and erosion (Figure 5B, W6). The dermatitis scores showed that the diseases were provoked with similar severity in all mice at W4, but the score decreased to different degrees depending on the treatment (Figure 5F). The GC-treated group (positive control) had the lowest dermatitis score, and the high FE-treated group was almost equal with no significance. The dermatitis scores of the low FE-treated group decreased significantly. The △TEWL represented an improvement of the skin barrier, and the △TEWL of the untreated group was a negative number indicating that the skin barrier was worsening. Similar to the dermatitis score, △TEWL improved with FE or GC treatment (Figure 5J).

The thickness of epidermal layers indicates AD. The untreated group showed 12.92-fold thicker epidermal layers than the healthy group (Figures 5C, G). The high FE group had a 3.48-fold thinner epidermal thickness than the untreated group (Figures 5C, G). A large number of mast cells is also a characteristic feature of AD, calculated by toluidine blue staining. The results revealed that the lowest infiltration of mast cells in the dermis was observed in the high FE- and GC-treated groups, with no significance. However, both were significantly superior to the untreated group (Figures 5D, H). Also, the retrieved skin was stained for detecting 8-OhdG, an oxidized product in DNA generated by the hydroxyl radicals (·OH) attack on the C-8 position of guanine (Prabhulkar and Li, 2010) (Figure 5E). The fluorescence intensity of 8-OHdG was increased remarkedly in the untreated AD groups. The levels of 8-OHdG decreased significantly compared with the untreated group after low and high FE treatments (Figure 5I), indicating that the reduction of oxidative stress in AD skin tissue was lowered by FE treatment.

Lastly, to study the immune mechanism of FE for AD treatment, the skin tissue in the lesion area was completely ground and crushed, and the acquired tissue fluid was tested for specific cytokine secretion to analyze the existence of functionally polarized CD4^+^ T-cells in AD (Romagnani, 1999). The IFN-γ, produced mainly by Th1 cells, was lowest in the high FE-treated group than in the untreated group. Low FE-treated and GC groups had decreased IFN-γ levels to some extent but to a lesser degree than the high FE-treated group (Figure 5K). IL-4, the Th2-mediated inflammatory cytokine, was reduced compared to the untreated group after high FE and GC intervention, respectively (Figure 5L). IL-13, another Th2-mediated inflammatory cytokine, showed a similar downward trend after a high FE or GC treatment (Figure 5M). Unfortunately, IL-17A-associated Th17 cells showed an association between groups (Figure 5N).

### 3.5 Immune mechanism of inhibition of Th2 cells differentiation by FE

**FIGURE 6:**
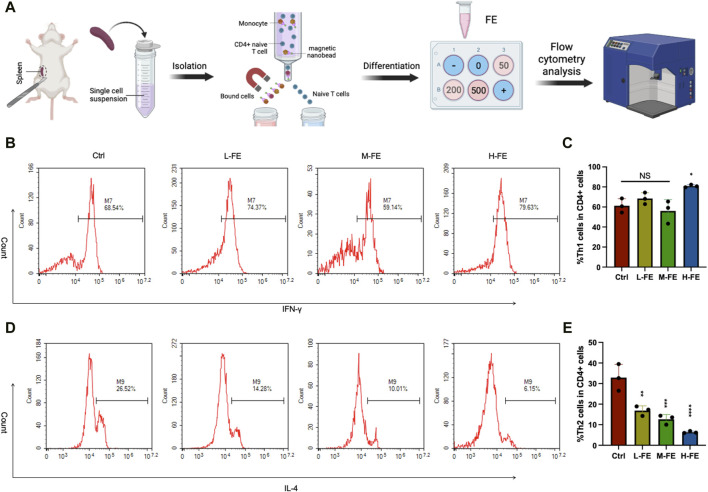
FE regulates the immune balance of Th1/2 cell **(A)** Schematic images of *in vitro* experiments with the mediation of Th cell differentiation by FE. Flow cytometry analysis of Th1/2 cells balance after incubating with various doses of FE. CD3^+^CD4^+^ cells and IFN-γ^+^ cells were gated for **(B)** Th1 cells, and **(D)** IL-4 cells were gated for Th2 cells. Th cells differentiation *in vitro* was repeated three times, and quantitative analysis of **(C)** Th1 cells showed no significance among groups. There was a remarkable downward trend of **(E)** Th2 cells as the dose of FE increased. (*p < 0.05, **p < 0.01, ***p < 0.001, ****p < 0.0001).

Th2 cell differentiation is considered to be the primary immune response that mediates AD (Böhm and Bauer, 1997). To explore the mechanisms of how FE improves AD symptoms, the differentiation of Th2 cells under FE culture was studied (Papandreou et al., 2020) (Figure 6A).

According to the standard T helper cell differentiation procedure of CD4^+^ T-cells *in vitro* (Sekiya and Yoshimura, 2016), Th2 cell differentiation is significantly reduced compared with that in the control group. Furthermore, increasing FE concentration in the culture medium further inhibits Th2 differentiation (Figures 6D, E). However, the various concentration FE treatments did not affect Th1 cell differentiation (Figures 6B, C). These results suggested that FE may improve AD symptoms by inhibiting the differentiation of Th2 cells.

## 4 Discussion

Atopic dermatitis (AD) is a chronic, relapsing inflammatory skin disorder that negatively impacts the patient’s quality of life. First-line treatment like TCSs has some disadvantages, such as skin atrophy, rosacea-like dermatitis, etc. Recent studies have found that a mesenchymal stem cell (MSCs)-based therapy has the potential to be used in AD and has no TCSs-related side effects (Lee et al., 2019; Daltro et al., 2020; Ryu et al., 2020; Bellei et al., 2022). However, stem cell-based therapy has some inevitable disadvantages, such as *in vitro* culture, immune response, and a high economic burden. Current research focuses on retaining the advantages of MSC therapy while improving its limitations. A recent study has unveiled that the therapeutic efficacy of mesenchymal stem cells (MSCs) arises primarily from the diverse array of bioactive factors secreted by MSCs, which exert regulatory control over various biological signaling pathways. Choi and Yu et al. found that MSCs extracted from Wharton’s jelly umbilical cord and its exosomes lowered the level of Th1, Th17, and Th2 produced by skin resident T-cells in an aspergillus fumigatus-induced AD mouse model (Song et al., 2019). More interestingly, the exosome-treated group demonstrated greater therapeutic efficacy than the MSCs-treated group, with less hypergranulosis and psoriasiform epidermal hyperplasia. Passing bioactive factors produced by MSCs is comparable to skimming the cream and removing impurities. FE derived from human adipose tissue has recently attracted attention in tissue engineering and regenerative medicine research as a notable example of a cell-free MSC-based strategy (Wang et al., 2020; Yin et al., 2020; Xu G-Y. et al., 2022; Xu M. et al., 2022). This research reported for the first time that FE, the cell-free fat extract, showed benefits in treating chronic refractory and recurrent dermatosis.

However, cells in the high FE group with a 500 μg/mL concentration showed negative proliferation on day 2 (Figure 2B). It is possible that FE had a positive effect only in the appropriate concentration range and that an overdose could cause cytotoxicity. Coincidentally, the apoptosis level in the H-FE group was marginally higher than in the M-FE group but significantly lower than that induced by ROS. It was reasonable to hypothesize that at the beginning intervention of FE, it first acted as a ROS scavenger to reverse apoptosis. Then an overdose of FE could affect cell viability.

Excessive levels of ROS can adversely affect cells’ lipid peroxidation, protein denaturation and DNA damage associated with many inflammation diseases and cancer. Recent evidence suggests that elevated ROS level is associated with the occurrence and progression of AD (Ji and Li, 2016; Lingappan, 2018; Borgia et al., 2022). Through analyzing the correlation between oxidative stress markers and clinical parameters, it has been confirmed a high oxidative burden in AD patients (Galiniak et al., 2022). It is believed that antioxidants would be a novel therapeutic method for AD. However, considering the uncertainty about accumulation toxicity of nanoparticles, we did not pre-clinically explore the use of nanomaterials with high ROS scavenging capacity (Bi et al., 2023; Im et al., 2023). FE, as a homologous biodrug without any cellular components, is barely non-immunogenic (Yu et al., 2018). A previous study demonstrated that FE has no toxic side effects on neuronal cells, but also significantly improves neuronal cell morphology, increases neurites length and actin intensity. In animal experiments on spinal cord injury, not only did no immune rejection occur, but also significantly reduced collagen formation and inflammatory cell infiltration, thus providing a favorable environment for nerve repair (Xu G-Y. et al., 2022). Additionally, FE already has recruiting and ongoing clinical trials (like NCT05883293, and NCT04311749).

According to our results, FE’s *in vivo* and *in vitro* ROS scavenging capability has been enhanced. In the DNCB-induced AD mouse model, the dermatitis score and TEWL, which represents the skin barrier state, significantly recovered (Figures 5G, K). For histological analyses and immunofluorescence of 8-OhdG, treatment with a high dose of FE significantly improved epidermis thickening, mast cell infiltration, IgE level, and oxidative stress damage. These results are similar to those of the positive control group with TCS. Still, extensive long-term use of corticosteroids usually leads to local and systemic complications, whereas FE does not and is expected to be developed as an AD daily care agent. Within the spectrum of inflammatory dermatoses, it is well-known that the ROS generated in skin lesions is related to the production of inflammatory cytokines, like IL-4, secreted by functional Th2 cells (Frossi et al., 2008). According to the analysis of specific cytokines in skin lesion tissues (Figures 5K–N), the inflammatory Th2-related cytokines (IL-4 and IL-13) significantly decreased. Similarly, Cho and collaborators found that Th2 cytokines in skin lesions were reduced under intravenous or subcutaneous administration of ADSC-exosomes (Cho et al., 2018). Notably, the inhibition effect was comparable to TCS at high FE doses. Thus, FE could inhibit the differentiation of CD4^+^ naïve T cells into Th2 cells and restore the Th1/2 cell balance. *In vitro*, we produced CD4^+^ naïve T cells that underwent differentiation upon introduction of various concentration FE. As FE concentration increased, the proportion of differentiated Th2 cells decreased significantly (Figures 6D, E). We hypothesized that is the result of inhibition of the JAK-STAT3/6-GATA3 pathway after the reduction of IL-4 and IL-13, thereby regulating T cell proliferation and Th2 cell differentiation (Gandhi et al., 2016; Zheng et al., 2023).

In addition, compared to a similar product, Platelet-rich plasma (PRP), which comes from blood, FE has the potential to break the limitations of autologous production for own use. That is standardized preparation of FE from medical waste emulsified fat and commercial availability for other people. However, if applying FE commercially, more strict consideration must be given (Atiyeh et al., 2021). For plastic and cosmetic surgery, exogenous growth factors promote soft tissue regeneration to achieve a desired filler effect for cosmetic facial rejuvenation or treatment of acne scars. But there are often many uncontrollable complications, like trigger tumor-like cell proliferation and immune inflammation (Long et al., 2024). And injection of exogenous growth factors into soft tissues is currently prohibited in China. FE contains a variety of highly concentrated growth factors, and it is crucial to standardize the concentration range that is safe and effective. Since we consider FE as a biodrug with a collection of factors, we opt to directly measure the protein content of FE rather than specifically analysing a particular growth factor. The results we found in our cellular experiments run counter to the report of Deng et al. (2020) that high concentrations (500ug/mL) have an inhibitory effect on cell viability after 72 h of co-incubation. AD always goes with skin barrier damege, leading to a rise in TEWL and resulting in irritating dryness and pruritus. Despite the discomfort, scratching persistently would further dysfunction skin barrier. In terms of therapeutic delivery, the development of a cream, ointment, or hydrogel dressing enriched with appropriate concentration of FE holds immense promise as a daily caring treatment for AD patients, offering a soothing and anti-inflammatory protective layer to alleviate their symptoms.

## 5 Conclusion

In conclusion, we first reported that FE, a cell-free human fat extract, has excellent antioxidant and ROS-scavenging properties. Additionally, FE can alleviate intradermal damage caused by excessive oxidative stress, as well as inhibit the differentiation of Th0 cells to Th2 cells. FE was also shown to well inhibit Th2-mediated inflammation, the specific manifestation of AD, which may be triggered by a decrease in IL-4 factor due to a decrease in ROS levels. These results suggested that FE may have the potential as a future treatment option for AD.

## Data Availability

The raw data supporting the conclusion of this article will be made available by the authors, without undue reservation.
